# Low Dose-Rate Interstitial Brachytherapy in Soft Tissue Sarcomas

**DOI:** 10.1080/13577149977721

**Published:** 1999-09

**Authors:** Edward Rosenblatt, Netanel Meushar, Mark Eidelman, Abraham Kuten

**Affiliations:** 1 Radiotherapy Unit Department of Orthopedic Surgery Rambam Medical Center and Faculty of Medicine Technion–Israel Institute of Technology Haifa 31096 Israel; 2 Orthopedic Oncology Service Department of Orthopedic Surgery Rambam Medical Center and Faculty of Medicine Technion–Israel Institute of Technology Haifa Israel

## Abstract

*Purpose.* To assess the effectiveness of Ir-192 interstitial brachytherapy as an adjunct to wide local excision as a functionsaving strategy for soft tissue sarcomas.

*Subjects and methods.* From September 1993 to April 1998, 20 consecutive patients diagnosed with soft tissue sarcomas were treated with a combination of wide local excision and interstitial brachytherapy. In 16 patients brachytherapy was done as an intraoperative procedure, while in four, the implant was performed post-operatively under local anesthesia. Eleven of the 20 patients also received external beam radiotherapy following the implant.

*Results.* After a mean follow-up of 27 months (4–54) the local control rate for all 20 patients was 85% (17/20). In the 16 patients who had an intra-operative implant, local control was 94% (15/16). In the four patients who underwent a post-operative implant, local control was 50% (2/4). Actuarial 5-year survival was 90%. There were three cases (15%) of severe local complications.

*Conclusions.* Wide local excision followed by low dose rate intersitital brachytherapy have yielded a 85% local control rate in 20 patients with soft tissue sarcomas. Local control rates were higher when the implants were done as an intra-operative procedure than as a post-operative one.


*Sarcoma* (1999) 3, 101–105

ORIGINAL ARTICLE

### Low dose-rate interstitial brachytherapy in soft tissue sarcomas

EDWARD ROSENBLATT,[1] NETANEL MEUSHAR,[2] MARK EIDELMAN[2] & ABRAHAM KUTEN[1]

[1]*Radiotherapy Unit and* [2]*Orthopedic Oncology Service, Department of Orthopedic Surgery, Rambam Medical Center and Faculty of Medicine, Technion—Israel Institute of Technology, Haifa, Israel*

**Abstract**

*Purpose.* To assess the effectiveness of Ir-192 interstitial brachytherapy as an adjunct to wide local excision as a function-saving strategy for soft tissue sarcomas.

*Subjects and methods.* From September 1993 to April 1998, 20 consecutive patients diagnosed with soft tissue sarcomas were treated with a combination of wide local excision and interstitial brachytherapy. In 16 patients brachytherapy was done as an intraoperative procedure, while in four, the implant was performed post-operatively under local anesthesia. Eleven of the 20 patients also received external beam radiotherapy following the implant.

*Results.* After a mean follow-up of 27 months (4–54) the local control rate for all 20 patients was 85% (17/20). In the 16 patients who had an intra-operative implant, local control was 94% (15/16). In the four patients who underwent a post-operative implant, local control was 50% (2/4). Actuarial 5-year survival was 90%. There were three cases (15%) of severe local complications.

*Conclusions.* Wide local excision followed by low dose rate intersitital brachytherapy have yielded a 85% local control rate in 20 patients with soft tissue sarcomas. Local control rates were higher when the implants were done as an intra-operative procedure than as a post-operative one.

**Key words**: *Brachytherapy, soft-tissue sarcomas.*

### Introduction

Over the last three decades several strategies have been used to avoid limb amputation while preserving local control rates in soft tissue sarcomas of the extremities. These include wide *en bloc* resection of soft parts, also known as compartmental resection,[1–3] pre- or post-operative external radiation therapy,[4–6] regional intra-arterial infusion[7] or perfusion chemotherapy,[8] and brachytherapy[9–13]. Each of these treatment methods has advantages and limitations.

The role of local brachytherapy as an adjunct to radical surgery has been clearly established by several investigators including the pioneer experience of the group at Memorial Sloan-Kettering.[14–16]

In this paper we describe our experience with low dose rate interstitial brachytherapy in a series of 20 patients with soft tissue tissue tumors at various anatomical locations.

### Patients and methods

Between September 1993 and March 1998, 20 patients with soft tissue sarcomas were managed with wide local excision and low dose rate interstitial brachytherapy as part of their treatment strategy. Patient characteristics are presented in Table 1.

The selection criteria were that all patients with a soft tissue tumor who were found eligible for general

**Table 1**. *Patient characteristics*

| | |
|---|---|
| Total number of patients | 20 |
| Mean age (range | 53 (12–90) |
| Male/female ratio | 8/12 |
| Adults (>15 years old) | 18 |
| Pediatric (<15 years old) | 2 |
| Limbs | 13 |
| Trunk | 5 |
| Head and neck | 2 |
| **Histologic types** | |
| Malignant fibrous hystiocytoma (MFH) | 5 |
| Liposarcoma | 1 |
| Leiomyosarcoma | 6 |
| Fibrosarcoma | 2 |
| Hemangiopericytoma | 2 |
| Malignant peripheral nerve sheath | 1 |
| Extraskeletal Ewing sarcoma (PNET) | 1 |
| Chondroid syringoma | 1 |
| Aggressive fibromatosis | 1 |

Correspondence to: Edward Rosenblatt, Radiotherapy Unit, Department of Oncology, Rambam Medical Center, Haifa 31096, Israel. Tel: +972 4 8543154; Fax: +972 4 8542929; E-mail: e_rosenblatt@rambam.health.gov.il

1357-714X print/1369-1643 online/99/020101-05 © 1999 Taylor & Francis Ltd anesthesia and a major surgical procedure, and were found compliant to undergo LDR brachytherapy under radiation precaution measures were selected. All patients received an explanation of the procedures, and informed consent for surgery and radiotherapy was obtained. There were eight males and 12 females; mean age was 53 years (range, 12–90). In 13 patients the tumor was located in the limbs (right arm, two left arm, two; right forearm, one; right gluteal, one; left gluteal, one; right thigh, two; left thigh, two; left groin, one; left leg one), while in five it was in the trunk and in two in the head-and-neck region, including one patient with a leiomyosarcoma of the tongue. Two patients were in the pediatric age group.

There were nine patients with stage I tumors (T1a–1b, T2a, N0), seven stage II (T2b, N0) and four stage III patients.[17] The grading system used was a two-grade system (low grade, G1 and G2, well and moderately differentiated; and high grade, G3 poorly differentiated and G4, undifferentiated) as recognized by the AJCC and as incorporated into the TNM stage grouping.[17] Following this classification there were four low-grade tumors (desmoid tumor, mixed liposarcoma, low-grade fibrosarcoma and a chondroid syringoma of the subcutaneous tissue). There was one case of extraskeletal Ewing's sarcoma/PNET of the anterior abdominal wall.

In 16 patients the brachytherapy was done as an intra-operative procedure immediately following wide local excision of the tumor or of the tumor bed after a marginal resection. These 16 patients were initially seen in a consultation before surgery/brachytherapy at a multidisciplinary sarcoma clinic including surgeons (N.M., M.E.) and radiation oncologists (E.R., A.K.). In the remaining four patients, brachytherapy was done post-operatively in patients previously resected with inadequate margins. These four patients were referred to our clinic following a surgical procedure at another hospital.

*Intra-operative brachytherapy procedures*

Elective wide excision and intra-operative brachytherapy were recommended in all 16 patients seen by us following a biopsy or marginal excision. Post-operative brachytherapy was done in the four patients operated elsewhere and in whom it was felt that an additional surgical procedure could not be done. In the initial nine patients of this series, and following the experience of Harrison *et al.*[16] and Gerbaulet *et al.*,[20] we attempted to give all the radiotherapy dose with an implant alone. We observed three cases of severe wound complications following the implant (see Results) and thus we changed our treatment policy giving only a third of the radiotherapy dose with the implant, and the additional two thirds by external beam techniques.

Under general anesthesia a wide local excision was performed dissecting through the healthy tissue surrounding the tumor except in areas where it came into close proximity with bone or with the neurovascular bundle of the limb. In these areas the tumor was peeled from these structures and brachytherapy catheters were placed above these high-risk regions.

Following tumor resection, gloves and instruments were changed. After the surgeon completed the tumor resection the tumor bed was marked with metallic clips for radiological identification. Under direct visualization a decision was made on the area to be implanted, attempting to cover the tumor bed with an additional 2 cm beyond the actual tumor confines. A series of parallel catheters were percutaneously inserted into the target area, placed approximately 1 cm apart and secured in proper position using catgut sutures as required. The entrance and exit points of the catheters were kept at not less than 1.5 cm from the surgical wound (Fig. 1). The wound was closed by approximation of soft tissues and skin over the catheters, care being taken to leave a drainage tube and thick viable flaps. Plastic and metallic brachytherapy buttons were anchored to the skin with loose stitches at the entrance and exit sites of the implant.

Five to 7 days after the surgical procedure, a pair of orthogonal radiographs of the implant, loaded with dummy metal sources, was obtained for source localization and computerized treatment planning.

The implant was then loaded with iridium-192 radioactive sources with an activity of 1.8 mCi/seed (BEST Industries, Inc., Springfield, VA) not earlier than the fifth post-operative day. The median tumor bed implant dose was 3300 cGy (1800–4900) delivered over 2–5 days. The dose was prescribed to an isodose located approximately 0.5 cm from the plane of the sources. This nominal prescribed dose represents the minimal dose within the target volume, the dose between or nearer the sources being significantly higher.[18] In 11 of 20 patients the interstitial implant was used to deliver part (approximately one-third) of the irradiation dose. In these patients the median implant dose was 2300 cGy (1800–3000). In nine of 20 patients the implant was the sole radiotherapy modality. Hence, in this

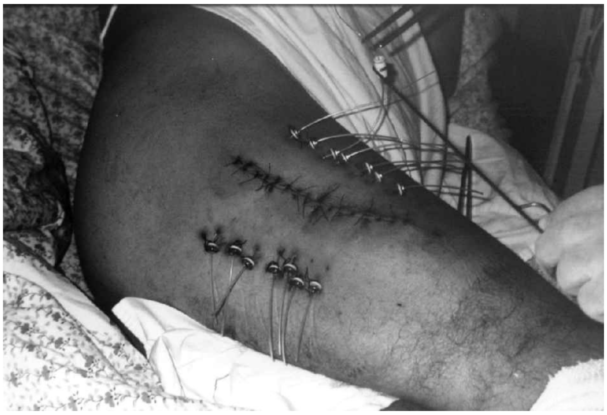

**Fig. 1.** *Single plane interstitial implant for a sarcoma of the right leg following wide local excision. Note entrance and exit sites of catheters far from the surgical wound. Plastic and metal buttons have been sewn to the skin with loose stitches.*

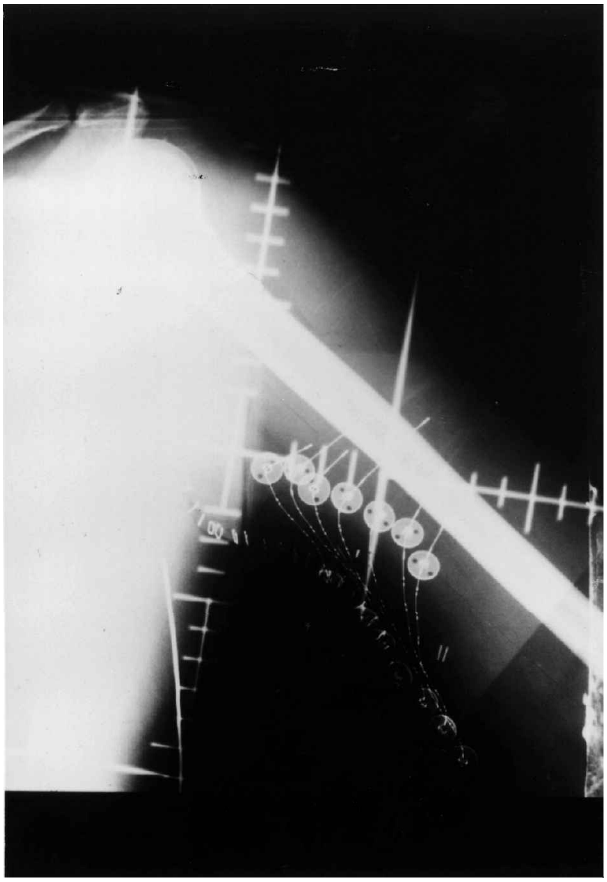

**Fig. 2.** *Radiograph of a single plane implant in the left arm with dummy metal sources for localization and dosimetry.*

group of patients the median implant dose was 4550 cGy (4500–4900). The variations on the prescribed dose depended on the assessment of clinical variables such as: anatomical site and proximity to sensitive structures, prior irradiation, geometric quality of the implant and potential risk for wound complications. When the implant plane was relatively close to the skin or neurovascular bundle, the lower dose (4500 cGy) was prescribed. In one patient with a hemangiopericytoma of the left groin resected with a positive margin, 4900 cGy was prescribed. This patient developed a local recurrence 3 years following the implant. The average implant dose rate was 48 cGy/h.

Eleven of 20 patients (55%) also received supplementary external beam irradiation to a median dose of 3920 cGy (1620–4500). No patient received post-operative adjuvant chemotherapy. In the two pediatric patients, chemotherapy was given before the surgical brachytherapy procedure. A 15-year-old boy with a low-grade fibrosarcoma of the neck received Cisplatin concomitantly with external irradiation before surgery/brachytherapy. A 12-year-old girl with a high-grade fibrosarcoma of the left thigh received three courses of ifosfamide–etoposide–mesna before surgery without response. In four patients the brachytherapy implant was performed as a separate procedure following a surgical resection with positive or close margins. This was done under local anesthesia and using brachytherapy needles as guides for the nylon tubes in the usual manner.

### Results

After a mean follow-up of 27 months (4–54) the local control rate for all 20 patients was 85% (17/20). Actuarial 5-year survival was 90%. Two patients died; one of heart failure and the other of sepsis following infection of the surgical wound.

Three patients eventually had failure at the implant site. Local recurrences presented at 2, 3 and 4 years following brachytherapy. One patient developed lung metastasis, being locally controlled at the primary tumor site.

In the 16-patients who had an intra-operative implant local control was 94% (15/16); in the four patients who underwent a post-operative implant local control was 50% (2/4). Table 2 presents local control rate as a function of various treatment variables.

### *Complications*

All patients developed variable degrees of erythema of the skin overlying the implant site 7–10 days following brachytherapy. This subsequently subsided.

Eleven patients (55%) developed chronic radiation changes at the implant site (NCI-CTC grade 2–3 toxicity). This changes consisted of dryness of the skin, subcutaneous fibrosis and telangiectasia. In several patients, these chronic radiation changes coexisted with cosmetic defects due to the surgical resection of a variable amount of soft tissue. Six of 11 patients with chronic radiation changes received an implant as the sole modality of radiotherapy, while in five patients the implant (to a lower dose) was followed by external beam irradiation.

We observed three cases of severe local complications. An 84-year-old diabetic patient with a large recurrence of MFH in her right gluteal area developed a wound infection and died of septic shock 2 months following surgery and brachytherapy.

A 12-year-old girl with a high-grade fibrosarcoma of the right thigh which did not respond to induction

**Table 2.** *Local control rates according to various clinical and treatment variables*

| Variable | *N* | Locally controlled |
|---|---|---|
| Adults | 18 | 15 |
| Children | 2 | 2 |
| Limbs | 13 | 12 |
| Trunk/head and neck | 7 | 5 |
| Intra-operative implant | 16 | 15 |
| Post-operative implant | 4 | 2 |
| Brachytherapy + EBRT | 11 | 11 |
| Brachytherapy alone | 9 | 6 |
| Adequate margins | 15 | 15 |
| Inadequate margins (< 5 mm or positive) | 5 | 3 |

chemotherapy developed progressive skin and subcutaneous necrosis, with subsequent delay in wound healing and knee contracture, in the months following treatment. This patient received 4500 cGy to the 0.5-cm plane with brachytherapy alone. Soft tissue necrosis was controlled with hyperbaric oxygen therapy and the knee function impairment required prolonged rehabilitation.

One patient developed a local abcess which resolved with incision and drainage.

### Discussion

Interstitial brachytherapy with Ir-192 as an adjuvant to radical surgery has already been shown to provide good local control rates in soft tissue sarcomas.[9–16] The use of brachytherapy as an adjuvant to surgery is based on its theoretical and practical advantages over external beam irradiation. The brachytherapy catheters are inserted in the tumor bed under direct visualization by the surgeon and the oncologist. Therefore, the high radiation dose is given to a target volume which encompasses the area with the greatest risk of containing residual microscopic disease. Because of the rapid fall-off of the dose with distance, the surrounding tissues are relatively spared and the use of low dose rate sources provides for a greater sparing of normal tissues.

In a prospective randomized trial that included 164 patients, Harrison *et al.*[16] obtained a local control rate of 82% with complete resection followed by Ir-192 brachytherapy, compared to 69% with surgery alone. In this trial the improvement in local control was limited to patients with high-grade histology, while patients with low-grade tumors did not benefit. Adjuvant brachytherapy improved local control, but did not have any significant impact on distant metastases or tumor-related mortality.

The role of radiation therapy in low-grade tumors has not been clearly elucidated. For relatively small lesions that have been completely excised with negative margins, postoperative irradiation is usually not recommended since a substantial fraction of these patients are cured by the surgical procedure. In these lesions radiotherapy is usually reserved for patients with positive margins, deep lesions that are difficult to follow or questionable margins in a location in which a local recurrence would require amputation. However, histologically low-grade tumors like fibrosarcoma grade I (aggressive fibromatosis, desmoid, dermatofibrosarcoma protuberans) have a typical 50% local recurrence rate following adequate surgery alone.[19]

In the present series brachytherapy was used in three patients with low-grade lesions: one with mixoid liposarcoma, a low-grade fibrosarcoma of the neck in a 15-year-old and a case of desmoid tumor (fibrosarcoma grade I) that recurred for the third time. In these three patients local control was obtained.

The Memorial experience in the early years of their trial[16] showed more major wound complications (11 of 23 patients) when the implants were loaded within the first 5 post-operative days, than when the loading was done after the fifth post-operative day (three of 21 patients). Following these results we loaded our implants not earlier than the fifth post-operative day. This timing allows the proliferative phase of wound healing to proceed without being impaired by radiation-induced reduction in fibroblast populations. We observed severe wound complications in two cases with probable predisposing factors. An 84-year-old patient with diabetes and ischemic heart disease was treated for a large (6×9×11 cm) recurrence of high-grade MFH in her gluteal region. This patient remained bedridden after the surgery/brachytherapy procedure. She subsequently developed wound infection and died of sepsis.

A 12-year-old girl with a high-grade fibrosarcoma of the thigh had previously received three courses of ifosfamide–etoposide–mesna combination chemotherapy without response. In this patient a wide local excision of the tumor was followed by full-dose brachytherapy (4500 cGy) to the tumor bed. The patient's age and previous chemotherapy may have been predisposing factors for the local complication. In the largest published series of low dose rate brachytherapy in children Gerbaulet *et al.*[20] treated 45 children giving prescribed doses of 6000–7500 cGy (Paris system). After a mean follow-up of 5 years, 78% of the patients were alive with no evidence of disease. A severe complication rate of 18% was observed in six of 33 evaluable patients. These six children received doses between 5800 and 7500 cGy to the prescription isodose, and in these cases, sequelae can probably be related to the high doses delivered with the implants. Being essentially a local modality treatment, brachytherapy success rates should be assessed in terms of local control. Current results are showing benefits for this radiotherapy modality in intermediate- and high-grade sarcomas following wide local excision. However, it is still unclear whether brachytherapy should be used alone or in combination with external beam techniques: and, if brachytherapy can be used alone, what would be the minimal effective dose to maintain current local control rates while minimizing wound complications? We feel that these two questions should be addressed and answered in future clinical trials.

In addition, there is an ongoing debate about the impact of local control on the development of distant metastases and disease-specific survival in patients with soft tissue sarcomas.

The importance of obtaining negative margins in the resection of soft tissue tumors is well established. It is noteworthy that our three patients who failed locally had been resected with inadequate margins (<5 mm or positive), while we noticed no local recurrences in the 15 patients who had pathologically adequate margins (>5 mm) (Table 2).

We strongly believe that successful results of this treatment approach depend on attention to technical details such as: wide resection with negative margins; marking of the tumor bed with metal clips; adequate coverage of the tumor bed and margins with the implant; loading of the implant not earlier than the fifth post-operative day; careful dosimetry; and dose prescription tailored to the clinical situation.

### References

1 Bowden L, Booher RJ. The principles and techniques of resection of soft tissue parts for sarcomas. *J Clin Oncol* 1987; 5:480–8.
2 Enneking WF, Spanier SS, Malawer MM. The effect of the anatomic setting on the results of surgical procedures for soft tissue parts sarcoma of the thigh. *Cancer* 1981; 47:1005–22.
3 Shiu MH, Castro EB, Hajdu SI, Fortner JG. Surgical treatment of 297 soft tissue sarcomas of the lower extremity. *Ann Surg* 1975; 182:597–602.
4 Lindberg R, Martin R, Ramsdahl M, Barkley HT. Conservative surgery and postoperative radiotherapy in 300 adults with soft tissue sarcoma. *Cancer* 1981; 47:2391–7.
5 Suit HD, Russell WO, Martin RG. Sarcoma of soft tissue: clinical and histologic parameters and response to treatment. *Cancer* 1975; 35:1478–3.
6 Suit HD, Proppe KH, Mankin HJ, Woods WC. Preoperative radiation therapy for soft tissue sarcomas. *Cancer* 1981; 47:2269–74.
7 Eilber FR, Mirra JJ, Grant TT. *et al.* Is amputation necessary for sarcomas? A seven year experience with limb salvage. *Ann Surg* 1980; 192:431–7.
8 Krementz E, Carter RD, Sutherland CM, Hutton EN. Chemotherapy of sarcomas of the limb by regional perfusion. *Ann Surg* 1977; 185:555–64.
9 Hilaris BS. *Handbook of interstitial brachytherapy*. Acton, MA: Acton Publishing Science Group, 1975.
10 Hilaris BS, Nori D, Andreson LL, eds. *Atlas of brachytherapy*. New York: McMillan, 1988.
11 Shiu MH, Turnbull AD, Nori D. *et al.* Control of locally advanced extremity soft tissue sarcoma by function-saving resection and brachytherapy. *Cancer* 1984; 53:1385–92.
12 Shiu MH, Colin C, Hilaris BS. *et al.* Limb preservation and tumor control in the treatment of popliteal and antecubital soft tissue sarcomas. *Cancer* 1986; 57:1632–9.
13 Shiu MH, Brennan MF, eds. *Surgical management of soft tissue sarcoma*. Philadelphia, PA: Lea & Febiger, 1989.
14 Hilaris BS, Shiu MH, Nori D. Limb-sparing therapy for locally advanced soft-tissue sarcomas. *Endocuriether Hypertherm Oncol* 1985; 1:17–9.
15 Shiu M, Hilaris B, Harrison L, Brennan M. Brachytherapy and function saving resection of soft tissue sarcoma arising in the limb. *Int J Radiat Oncol Biol Phys* 1991; 21: 1485–92.
16 Harrison LB, Franzese F, Gaynor JJ, Brennan M. Long-term results of a prospective randomized trial of adjuvant brachytherapy in the management of completely resected soft tissue sarcomas of the extremity and superficial trunk. *Int J Radiat Oncol Biol Phys* 1993; 27:259–65.
17 American Joint Committee on Cancer Staging Manual, 5th edn. Philadelphia, PA: Lippincott-Raven, 1997.
18 Rosenblatt E. Dose specification in Iridium-192 intersitital brachytherapy. In: *Radiation dose in radiotherapy, from prescription to delivery*. IAEA-TECDOC-734:91-101, 1994.
19 Sherman NE, Romsdahl M, Evans H. *et al.* Desmoid tumors: a 20 year radiotherapy experience. *Int J Radiat Oncol Biol Phys* 1990; 19:37–40.
20 Gerbaulet A, Panis X, Flamant F, Chassagne D. Iridium afterloading curietherapy in the treatment of pediatric malignancies. *Cancer* 1985; 56:1274–9.
